# Deep Learning-Based Image Steganography with Latent Space Embedding and Smart Decoder Selection

**DOI:** 10.3390/e27121223

**Published:** 2025-12-02

**Authors:** Yiqiao Zhou, Na Wang, Xiaolong Hong, Yanchun Peng, Shuo Shao

**Affiliations:** 1 School of Optical-Electrical and Computer Engineering, University of Shanghai for Science and Technology, Shanghai 200093, China; 2335060131@st.usst.edu.cn (Y.Z.); wna@usst.edu.cn (N.W.); 243360805@st.usst.edu.cn (X.H.); 2335061119@st.usst.edu.cn (Y.P.); 2 Department of System Science, University of Shanghai for Science and Technology, Shanghai 200093, China

**Keywords:** image steganography, deep learning, adaptive encoder–decoder framework, robustness to noise, steganalysis

## Abstract

Image steganography is crucial for secure communication, enabling covert data embedding within cover images. While traditional methods such as LSB embedding are vulnerable to detection, deep learning techniques like GANs and autoencoders have improved performance, yet they still struggle with dynamic adaptation to diverse secret data types, limited training datasets, and resilience to distortions. To address these issues, we propose a flexible framework with adaptive multi-encoder–decoder pairs, extensive dataset training, and an optimized architecture with specialized components. Our model achieves significant improvements in Secret Recovery Accuracy (SRA), Stego-Image Quality (SSIM, PSNR), and robustness to noise, with SSIM of 0.99 and recovery accuracy over 98%. It also reduces the detection rate, with an AUC approaching 0.5 in steganalysis. These results set a new benchmark for secure image transmission and privacy-preserving communication.

## 1. Introduction

Image steganography is a critical technology in modern digital communication, particularly for ensuring secure data transmission [1,2,3,4]. It allows secret information to be embedded within cover images in a way that is imperceptible to the human eye, enabling covert communication and securing transmission channels in scenarios where traditional encryption alone may be less effective or more vulnerable. In addition, image steganography underpins many digital watermarking applications, providing practical tools for copyright protection and content authentication [5]. However, conventional schemes, such as Least Significant Bit (LSB) embedding and frequency-domain transformations, are often susceptible to detection and lack the robustness needed to withstand advanced signal-processing attacks [4,6]. This gap between practical requirements and algorithmic resilience has motivated a growing demand for more sophisticated and robust steganographic techniques to address evolving security challenges [7,8].

The emergence of deep learning has substantially advanced image steganography, enabling more adaptive and powerful hiding strategies [1,5,9]. Generative Adversarial Networks (GANs) and autoencoders, in particular, have made it possible to directly learn joint encoding–decoding functions from large image collections [10,11,12,13]. GAN-based methods exploit the competition between a generator and a discriminator to improve the visual realism of stego images and reduce detectability, while autoencoder-based approaches learn compact latent representations that support efficient embedding and reliable recovery [9,14,15]. These deep learning-based techniques alleviate several limitations of traditional methods, including limited embedding capacity and poor robustness to common image manipulations or compression, thereby broadening the application scope of secure, hidden communication [11,16].

Despite these advances, a key practical challenge remains in what may be termed mechanistic overfitting. Many contemporary systems still rely on a single, task-agnostic encoder–decoder pipeline that implicitly assumes that both the secret payloads and the transmission channel follow a relatively narrow distribution. In practice, the shared feature extractor tends to specialize to the statistics seen during training, so performance can deteriorate once the payload or channel conditions deviate from this regime. At the same time, many pipelines adopt a restricted view of the transmission channel: they are optimized under weak perturbations (often only mild Gaussian noise) or a single distortion model [17,18]; therefore, they degrade sharply under more realistic perturbation families, including JPEG quantization, impulse noise, complex sensor noise, blur, geometric warps, weather-like artifacts, and random erasing. Together, a structurally overfitted representation (tied to a single encoder–decoder path) and a channel-overfitted training regime (tied to a narrow perturbation model) help explain why current state-of-the-art models often struggle to retain both secrecy and perceptual fidelity once deployed in real acquisition, compression, and transmission chains [15,19,20].

In practical steganographic scenarios, secret images are highly heterogeneous in both semantics and statistics (scenery, faces, pets, logos, etc.), with very different texture, frequency, edge, and color patterns. Processing all such secrets with a single encoder–decoder leads to latent representations that favor dominant categories and generalize poorly under distribution shifts, i.e., mechanistic overfitting. To mitigate this, we introduce a secret pre-processing stage that standardizes each secret (including resizing to a fixed resolution) and extracts discriminative cues to assign a coarse semantic label (e.g., “scenery”, “face”, “pet”). This label is extensible as new categories are added and is used as an explicit routing signal to select a specialized expert path for hiding and recovery; in principle, any original secret size can be embedded via this standardized input, although aggressive downsampling of very large or highly detailed secrets inevitably limits the maximum reconstruction fidelity.

On the reconstruction side, the architecture is designed to separate secret-related information from cover content in the latent space: the cover is encoded into a cover feature, the resized secret into a secret feature, and the two are fused into a shared stego representation that is decoded along the single expert path selected by the semantic label. A dedicated secret decoder branch operates on this stego feature, and joint losses on cover fidelity, stego fidelity, and secret reconstruction encourage disentanglement between cover and secret information. The routing label is produced by a lightweight dual-branch selector that combines evidence from the fused stego feature and the (potentially noisy) stego image. Covers and secrets are drawn from disjoint pools, while the same heterogeneous secret pool is shared between the pre-processing classifier and the hiding/recovery modules, and the cover encoder is independent of the secret label, whereas the secret branch does not depend on the cover type. As a result, the method is intended to remain robust whether the cover and secret belong to the same semantic category or not, a behavior confirmed empirically on diverse datasets.

Extensive experiments on benchmark datasets, including ImageNet, MNIST, MetFACE, and Stock1K, were conducted to evaluate the performance of the proposed model. The evaluation considers key metrics such as Secret Recovery Accuracy (SRA), Stego-Image Quality (SSIM, PSNR), and resilience to image distortions. We analyze the controllable trade-off between cover imperceptibility and secret fidelity across different effective compression ratios (including the impact of resizing and payload size), robustness under various noise and corruption conditions, and comparisons with existing deep learning-based steganographic methods. When compared with models such as HiNet, InvMIHNet, and a baseline CNN autoencoder, the proposed framework achieves consistently competitive or improved PSNR, SSIM, and SRA, along with substantially reduced detection rates in steganalysis tests, with AUC values close to 0.5 in the evaluated setting. These results suggest that the framework provides both theoretical and empirical support for the design of robust image steganography systems for secure image transmission, digital watermarking, and privacy-preserving communication under a wide range of real-world distortions and data complexities.

The rest of this paper is organized as follows: Section 2 provides the preliminaries, including the key notations and performance metrics. Section 3 describes the proposed steganographic framework in detail, including the secret pre-processing, multi-path architecture, and training strategy. Section 4 presents the experimental results and compares the proposed approach with existing methods through ablation and comparative experiments. Finally, Section 5 concludes the paper and discusses potential directions for future research.

## 2. Preliminaries

This section introduces the essential mathematical concepts, key notations, and performance metrics used in our steganographic framework. We define the information capacity, along with performance metrics such as Peak Signal-to-Noise Ratio (PSNR), Structural Similarity Index (SSIM), Message Recovery Accuracy (ACC), and Area Under the Curve (AUC) that are integral to evaluating the quality of both the stego images and the recovered secret data.

The PSNR is a standard metric for evaluating the quality of the stego image by comparing it to the original cover image. It is defined as follows:

(1) $$\mathrm{PSNR}=10\cdot \log_{10}\left(\frac{MAX_{I}^{2}}{MSE}\right),$$

where $MAX_{I}$ is the maximum possible pixel value (usually 255 for 8-bit images), and MSE is the Mean Squared Error between the original image *X* and the stego image $\hat{X}$:

(2) $$MSE=\frac{1}{N}\sum_{i=1}^{N}{\left(X_{i}-{\hat{X}}_{i}\right)}^{2},$$

where *N* is the number of pixels, and $X_{i}$ and ${\hat{X}}_{i}$ are the pixel values of the cover and stego images, respectively. A higher PSNR indicates better imperceptibility, as the stego image is visually closer to the original cover image.

The SSIM is another important metric for measuring the perceptual similarity between the cover and stego images. It is defined as follows:

(3) $$SSIM\left(X,\hat{X}\right)=\frac{\left(2\mu_{X}\mu_{\hat{X}}+C_{1}\right)\left(2\sigma_{X\hat{X}}+C_{2}\right)}{\left(\mu_{X}^{2}+\mu_{\hat{X}}^{2}+C_{1}\right)\left(\sigma_{X}^{2}+\sigma_{\hat{X}}^{2}+C_{2}\right)},$$

where $\mu_{X}$ and $\mu_{\hat{X}}$ are the mean values of the cover and stego images, respectively; - $\sigma_{X}^{2}$ and $\sigma_{\hat{X}}^{2}$ are the variances of the cover and stego images, respectively; - $\sigma_{X\hat{X}}$ is the covariance between the cover and stego images; and - $C_{1}$ and $C_{2}$ are constants to stabilize the division.

SSIM evaluates the structural similarity between the cover and stego images. Higher SSIM values suggest that the stego image maintains more structural integrity, leading to higher imperceptibility.

The ACC quantifies the precision with which the secret message is recovered from the stego image. It is defined as the percentage of correctly recovered bits from the total number of embedded bits:

(4) $$\mathrm{ACC}=\frac{1}{L}\sum_{i=1}^{L}I\left(w_{i}={\hat{w}}_{i}\right),$$

where *L* is the length of the secret message, $w_{i}$ and ${\hat{w}}_{i}$ are the original and recovered secret bits, respectively, and I(·) is the indicator function, which is equal to 1 if the condition is true and 0 otherwise.

The ACC measures the ability of the system to recover the secret data accurately. Higher ACC values indicate better recovery accuracy, meaning the system is more reliable in retrieving the secret message.

The AUC is a standard metric used to evaluate the effectiveness of a steganographic system against steganalysis. It is particularly useful for assessing the detectability of the stego image by a classifier or detector. AUC measures the ability of a detection system to distinguish between cover and stego images. It is computed from the Receiver Operating Characteristic (ROC) curve, which plots the True Positive Rate (TPR), representing the fraction of correctly detected stego images, against the False Positive Rate (FPR), representing the fraction of cover images incorrectly identified as stego images.

The AUC is defined as the area under the ROC curve:

(5) $$\mathrm{AUC}=\int_{0}^{1}f\left(\alpha \right)\;d\alpha ,$$

where α represents the False Positive Rate (FPR), and f(α) is the corresponding True Positive Rate (TPR) as a function of the FPR.

Unless otherwise noted, AUC is measured for the steganalysis detector. Therefore, AUC ≈ 0.5 is desirable for the hider (good resistance), while AUC → 1.0 signifies that the detector is highly effective (poor resistance).

## 3. Proposed Steganographic Framework

### 3.1. Overview

**Figure 1:**
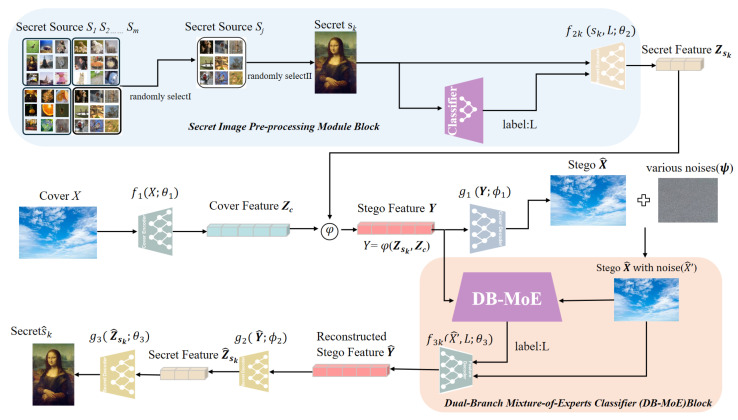
Overall System Architecture.

As depicted in Figure 1, the proposed framework first initiates with the pre-processing of the secret image, where salient features—such as texture, structure, and color distribution—are extracted and a semantic label is assigned to categorize the image (e.g., scenery, facial, or pet images). Following pre-processing, the secret data is embedded into the cover image, as the second part, through a multi-encoder–decoder architecture, employing specialized encoder–decoder pairs tailored to the specific characteristics of each secret data category. The encoder transforms the cover image into a latent space, where it is subsequently combined with the secret data and passed through the decoder, resulting in the stego image while minimizing perceptual distortion. Third, to simulate real-world distortions, noise is intentionally introduced to the stego image, enhancing the robustness of the framework against various attacks. Then, the noisy stego image is processed by a dual-parallel classifier: one classifier evaluates the high-level feature representation of the image, while the other operates directly on the noisy stego image itself. The outputs of both classifiers are integrated and passed to the decoder, which refines the secret recovery process. Eventually, the decoder reconstructs the original secret data with high fidelity, even in the presence of noise. This cohesive workflow facilitates efficient data embedding and recovery, maintaining imperceptibility while ensuring resilience to a wide range of distortions.

In the following subsections, we will focus on three pivotal innovations of our model: the secret image pre-processing module, the bicameral consensus classifier, and the multi-encoder–decoder architecture with static and dynamic mechanisms. These components are integral to the framework’s ability to achieve high-quality, imperceptible steganography and robust secret recovery.

### 3.2. Secret Image Pre-Processing Module

At the outset, the secret image undergoes a sequence of pre-processing steps (as illustrated by the blue-highlighted block in Figure 1) to enhance the system’s robustness and adaptability. First, a secret image is randomly selected from heterogeneous sources—natural scenes, facial photographs, or pet pictures—and resized to a fixed spatial resolution compatible with the network, so that all subsequent modules operate on a standardized secret tensor regardless of the original image size. Next, a lightweight classifier extracts discriminative features (structure, texture, color distribution, edge density, and spatial-frequency content) and, based on these cues, assigns a semantic label from the predefined classes: Category 0—Scenery (e.g., landscapes, architecture, sky), Category 1—Facial Appearance (e.g., portraits, face photographs), and Category 2—Pet (e.g., animal photographs). These classes are characterized by distinct visual attributes: scenery images typically exhibit abundant texture and high spatial frequencies; facial images show pronounced anatomical features and relatively smooth skin; and pet images present distinctive shapes, colors, and fur textures. Importantly, the scheme is extensible and can be augmented with additional categories (e.g., object, indoor scene), with the classifier readily adaptable to such additions.

In simple terms, this pre-processing module—beginning the overall pipeline—forms a coherent sequence of selection → classification → category-specific encoding, ensuring proper categorization and standardization while strengthening the framework’s robustness and generalizability.

Specifically and mathematically, before starting the embedding process, we first consider the set of *m* candidate secret sources. Let each secret source be denoted as $S_{j}$ for j=1,…,m. Each secret source $S_{j}$ contains a set of secret images, which we denote as $S_{j}=\left\{s_{j1},s_{j2},\ldots ,s_{jn_{j}}\right\}$, where $n_{j}$ is the number of secret images in source $S_{j}$. We first randomly select one secret source $S_{j}$ from the set of *m* sources. Then, from the selected source $S_{j}$, a secret image is randomly chosen. We denote this image by $s_{k}\in S_{j}$, where *k* is the index of the selected secret image.

(6) $$\begin{array}{cc} S_{j} & \in \{S_{1},S_{2},\ldots ,S_{m}\},\\ j & ∼\mathrm{Uniform}(1,\ldots ,m). \end{array}$$

The selected secret image is then resized to a fixed spatial resolution by a deterministic operator R:

(7) $${\tilde{s}}_{k}=R\left(s_{k}\right),$$

where ${\tilde{s}}_{k}$ has the canonical height and width required by the network.

Next, a lightweight classifier C assigns the resized secret image ${\tilde{s}}_{k}$ to one of *K* semantic classes:

(8) $$C:\;X\to K=\left\{1,\ldots ,K\right\},\;c_{k}\;=\;C\left({\tilde{s}}_{k}\right),$$

where $c_{k}$ is the class label of $s_{k}$, determined by characteristic structure, texture, color distribution, edge density, and spatial-frequency content.

- Input: The set of secret sources $\left\{S_{1},S_{2},\ldots ,S_{m}\right\}$ and their respective secret images.
- Process: Randomly select a secret source $S_{j}$ as in (6), and then draw a secret image $s_{k}\in S_{j}$, resize it via (7) to obtain ${\tilde{s}}_{k}$, and compute its class label $c_{k}=C\left({\tilde{s}}_{k}\right)$ as in (8).

After classification, one secret image is selected for embedding based on the downstream policy (e.g., a required class or sampling rule). Let ${\tilde{s}}_{k}$ denote its resized version as in (7); by abuse of notation, we define the standardized secret input used for hiding as

(9) $$S\;\leftarrow \;{\tilde{s}}_{k}.$$

- Input: The selected (and resized) secret image ${\tilde{s}}_{k}$.
- Process: Use $S\leftarrow {\tilde{s}}_{k}$ in (9) as the standardized secret image for subsequent embedding into the cover.

As part of the secret image pre-processing module, the selected secret image *S* and its corresponding label *L* are fed into a dedicated encoder. This encoder, denoted as $f_{2k}\left(S,L;\theta_{2}\right)$, processes both the secret image and its label to generate a feature representation $Z_{sk}$. The function $f_{2k}$ is parameterized by $\theta_{2}$, which represents the set of model parameters learned during training. By incorporating the label *L* (typically taken as $c_{k}$), the encoder can produce a more accurate and context-aware feature representation. The output feature $Z_{sk}$ is then used in the subsequent stages of the framework for embedding into the cover image. This process ensures that the model efficiently handles secret data by transforming it into a compact and discriminative feature representation, while preserving the essential information for embedding:

(10) $$Z_{sk}=f_{2k}\left(S,L;\theta_{2}\right).$$

Although the mapping C in Equation (8) is implemented as a lightweight CNN classifier, its role in our framework goes beyond standard image categorization. In a conventional setting, classification is typically used only to assign a semantic tag to an image. In contrast, in our design, the predicted label $c_{k}$ serves as a task-aware control signal that (i) routes the secret image to a dedicated encoder–decoder expert tuned to its statistics, (ii) partitions the secret space according to low- and mid-level cues (texture, frequency content, edge density) that directly affect embedding capacity and robustness, and (iii) standardizes the secret representation before fusion with the cover feature. Because the same heterogeneous secret-image pool is used both to train the pre-processing classifier and to train the embedding and reconstruction modules, the decision boundaries of C are aligned with the regimes where different experts are most effective, rather than being optimized for classification accuracy alone. As a result, the pre-processing module does not merely recognize image content but actively shapes how model capacity is allocated across secret types and how robust the subsequent steganographic pipeline is to distribution shifts in the secret payloads.

In practice, Section 4.3 shows that this label-driven routing, together with the corresponding expert branches, yields consistently higher PSNR/SSIM and bit accuracy (and lower LPIPS) than a single-path variant without secret pre-processing or semantic labels. This comparison indicates that the classification-and-routing stage contributes materially to both hiding and reconstruction quality. Section 4.2 further demonstrates that the performance of the full model remains stable when the label space is expanded from three to five classes, supporting the extensibility of the proposed design.

### 3.3. Dual-Branch Mixture-of-Experts Classifier (DB-MoE)

**Figure 2:**
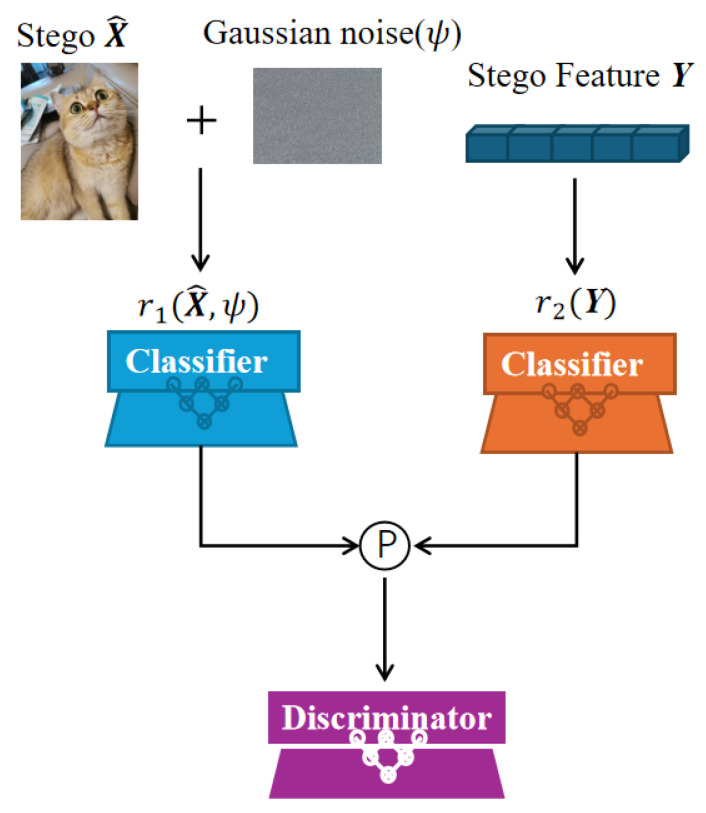
Dual-Branch Mixture-of-Experts Classifier (DB-MoE).

This module corresponds to the skin-toned block in Figure 1. Specifically, our DB-MoE model comprises a pair of parallel classifiers—a main classifier and an auxiliary classifier—and a discriminator, as shown in Figure 2. It implements a parallel, dual-branch classifier that operates within the cover–stego pipeline. The module takes two inputs: (i) the fused stego feature Y, obtained by feature fusion of the secret and cover; and (ii) a noise-processed stego image $\hat{X}$ produced by the noise module. The main branch consumes the high-level fused representation Y, while the auxiliary branch acts directly on the noise-processed stego $\hat{X}$. Each branch produces a score vector, and these scores are combined at the decision level to yield a single predicted label L. Finally, the predicted label L is forwarded—together with the stego-with-noise $\hat{X}$—to the downstream decoder, enabling conditioned decoding while preserving parallel, disentangled feature extraction and improving robustness.

Let Y denote the stego feature and $\hat{X}$ the stego image. We employ a main classifier $r_{2}$ acting on Y and an auxiliary classifier $r_{1}$ acting on $\hat{X}$ with parameters ψ. Both produce class logits in $R^{K}$:

(11) $$\begin{array}{cc} r_{1}\left(\hat{X};\psi \right) & =\mathrm{AuxiliaryClassifier}\left(\hat{X};\psi \right)\in R^{K},\\ r_{2}\left(Y\right) & =\mathrm{MainClassifier}\left(Y\right)\in R^{K}. \end{array}$$

We denote their outputs by

(12) $$\begin{array}{cc} z_{1} & =r_{1}\left(\hat{X};\psi \right),\\ z_{2} & =r_{2}\left(Y\right). \end{array}$$

Next, we fuse these logits by a weighted sum, where α and β are the fusion weights. These weights satisfy α+β=1, ensuring that the logits are combined in a balanced manner:

(13) $$z=\alpha \cdot z_{2}+\beta \cdot z_{1},\;\alpha ,\beta \geq 0,\;\alpha +\beta =1.$$

In our implementation, we use a fixed fusion ratio and set

(14) $$z=0.8z_{2}+0.2\;z_{1},$$

which corresponds to fixed coefficients α=0.8 and β=0.2 in Equation (12). These coefficients are not learned but, rather, chosen once as hyper-parameters and kept constant throughout training and evaluation.

The fused logits z are then passed through the softmax function to convert them into probabilities:

(15) $$p=\mathrm{softmax}\left(z\right),\;p_{k}=\frac{e^{z_{k}}}{\sum_{j=1}^{K}e^{z_{j}}},$$

where p is the probability distribution across the classes, and $p_{k}$ represents the probability of class $C_{k}$.

To obtain the final predicted label *L*, we take the class with the highest probability:

(16) $$L=\arg\max_{k\in \{1,\ldots ,K\}}p_{k},$$

where *L* is the predicted label (the class with the highest probability).

After that, we introduce the noise-processed stego image ${\hat{X}}^{\prime }$, which is obtained by adding Gaussian noise n to the stego image $\hat{X}$:

(17) $${\hat{X}}^{\prime }=\hat{X}+n,\;n∼N\left(0,\psi I\right),$$

where ψ represents the noise variance, and n is the Gaussian noise added to $\hat{X}$. Then, the predicted label *L* is used directly to condition the subsequent stego feature reconstruction. Since no one-hot encoding is used, the label *L* is passed as is to the decoder:

(18) $$\hat{Y}=f_{3k}\left({\hat{X}}^{\prime },L;\theta_{3}\right),$$

where $f_{3k}$ is the decoder that reconstructs the stego feature $\hat{Y}$ conditioned on the noisy stego image $\tilde{X}$ and the label *L*.

Finally, we can define the training objectives for this stage. The classifier loss $L_{\mathrm{cls}}$ is based on cross-entropy between the true label $L^{⋆}$ and the predicted probabilities p, and the feature consistency loss $L_{\mathrm{feat}}$ ensures that the reconstructed stego feature $\hat{Y}$ matches the original stego feature Y:

(19) $$L_{\mathrm{cls}}=-\log p_{L^{⋆}},$$

(20) $$L_{\mathrm{feat}}={∥\hat{Y}-Y∥}_{2}^{2},$$

where $L_{\mathrm{cls}}$ minimizes the classification error, and $L_{\mathrm{feat}}$ minimizes the reconstruction error for the stego features.

The total loss for this module is the weighted sum of the classifier loss and feature consistency loss:

(21) $$L_{\mathrm{total}}=\lambda_{\mathrm{cls}}L_{\mathrm{cls}}+\lambda_{\mathrm{feat}}L_{\mathrm{feat}},$$

where $\lambda_{\mathrm{cls}}$ and $\lambda_{\mathrm{feat}}$ are the loss weights for classification and feature consistency, respectively.

This dual-parallel classifier processes cover and stego inputs concurrently, thereby enhancing robustness and classification accuracy. The main branch operates on the fused, high-level representation derived from the cover–stego pair, while the auxiliary branch classifies directly from stego-specific cues. A weighted fusion of their outputs yields a balanced consensus, integrating complementary evidence from both branches.

### 3.4. Multi-Encoder–Decoder Embedding Module

In this subsection, we will introduce the multi-encoder–decoder process, where the cover image and the secret image are encoded and fused to produce the stego feature, which is then decoded to reconstruct the secret data. Then, we will delve into the training process, where the objective is to minimize the difference between the original and reconstructed stego features while also optimizing the extraction of secret features and their reconstruction. This involves the application of several loss functions and a dynamic loss adjustment strategy, which will be discussed in detail.

#### 3.4.1. Mathematical Formulation of the Multi-Encoder–Decoder Pairs

After the secret pre-processing block, we take as inputs the category label *L* and the secret feature $Z_{s_{k}}$ extracted from the selected secret image $s_{k}$. As shown in the white region of Figure 1, the first step is to pass the cover image *X* through the cover encoder $f_{1}\left(\cdot ;\theta_{1}\right)$, which generates a latent cover feature $Z_{c}$. The secret feature $Z_{s_{k}}$ is then fused with $Z_{c}$ through a fusion operator φ(·,·), producing the stego feature Y. The cover decoder $g_{1}\left(\cdot ;\phi_{1}\right)$ is used to reconstruct the stego image $\hat{X}$ from Y.

To introduce noise, a noise layer is applied to the reconstructed stego image $\hat{X}$, generating the noise-processed stego ${\hat{X}}^{\prime }$. This pair $\left\{Y,{\hat{X}}^{\prime }\right\}$ is then passed to the downstream Dual-Branch Mixture-of-Experts (DB-MoE) classifier (skin-toned block). For the purpose of this subsection, we focus on the output: after the DB-MoE module, we obtain a reconstructed stego feature $\hat{Y}$, which will be used by the secret-decoding stage (outside the scope of this subsection).

The multi-encoder–decoder part can be formulated as follows: First, the cover image *X* is encoded using the cover encoder $f_{1}\left(\cdot ;\theta_{1}\right)$ to produce the cover feature $Z_{c}$:

(22) $$Z_{c}=f_{1}\left(X;\theta_{1}\right),$$

where $f_{1}$ is a deep neural network encoder that maps the cover image to a feature space $Z_{c}$.

Next, we fuse the secret feature $Z_{s_{k}}$ and the cover feature $Z_{c}$ using the fusion operator φ(·,·) to produce the stego feature Y:

(23) $$Y=\varphi (Z_{s_{k}},Z_{c};\phi ).$$

This fusion operation, which could be concatenation, gated addition, or attention-based fusion, combines the cover and secret features to form a feature that carries the embedded secret data. After that, the cover decoder $g_{1}\left(\cdot ;\phi_{1}\right)$ is applied to the fused stego feature Y to reconstruct the stego image $\hat{X}$:

(24) $$\hat{X}=g_{1}\left(Y;\phi_{1}\right),$$

where $g_{1}$ decodes the stego feature back into the image space, generating a visually plausible stego image.

Then, we focus on the process of extracting the secret feature from the stego feature and subsequently decoding it to recover the secret image. The process begins with the extraction of the secret feature ${\hat{Z}}_{s_{k}}$ from the reconstructed stego feature $\hat{Y}$, which is performed through the secret decoder. This feature is then used in the decoding step to recover the original secret image ${\hat{S}}_{k}$.

The stego feature $\hat{Y}$ is passed through the secret decoder $g_{2}$, which extracts the secret feature ${\hat{Z}}_{s_{k}}$:

(25) $${\hat{Z}}_{s_{k}}=g_{2}\left(\hat{Y};\phi_{2}\right),$$

where $g_{2}$ represents the secret decoder function that extracts the secret feature ${\hat{Z}}_{s_{k}}$ from the given stego feature $\hat{Y}$. This step effectively isolates the secret information embedded within the stego image.

Next, the secret feature ${\hat{Z}}_{s_{k}}$ is decoded by the secret decoder $g_{3}$ to reconstruct the original secret image ${\hat{S}}_{k}$:

(26) $${\hat{S}}_{k}=g_{3}\left({\hat{Z}}_{s_{k}};\theta_{3}\right),$$

where $g_{3}$ is the decoder that reconstructs the original secret image ${\hat{S}}_{k}$ from the extracted secret feature ${\hat{Z}}_{s_{k}}$.

Because the cover encoder $f_{1}$ only processes the cover image *X* and never sees the raw secret, while the secret decoders $g_{2}$ and $g_{3}$ operate solely on the fused stego feature $\hat{Y}$ and its routed label, the model is explicitly encouraged to factor cover- and secret-related information into separate latent channels.

#### 3.4.2. Training Objective for Multi-Encoder–Decoder Feature Reconstruction

The training process now aims to minimize the difference between the reconstructed stego feature $\hat{Y}$ and the original stego feature Y. To achieve this, we use a feature consistency loss function, which is defined as follows:

(27) $$L_{\mathrm{feat}}=\gamma {∥\hat{Y}-Y∥}_{2}^{2},$$

where γ is a weight for the feature consistency loss. This ensures that the reconstructed stego feature $\hat{Y}$ remains close to the original feature Y, preserving the embedded secret data.

The next step in the process is to decode the secret from the reconstructed stego feature $\hat{Y}$. The training loss combines both the feature reconstruction loss and a classification loss $L_{\mathrm{cls}}$ (if applicable). The total loss for training is the weighted sum of these two losses:

(28) $$L_{\mathrm{total}}=\lambda_{\mathrm{cls}}L_{\mathrm{cls}}+\lambda_{\mathrm{feat}}L_{\mathrm{feat}},$$

where $\lambda_{\mathrm{cls}}$ and $\lambda_{\mathrm{feat}}$ are weights that balance the contributions of the classification and feature consistency losses during training.

**Figure 3:**
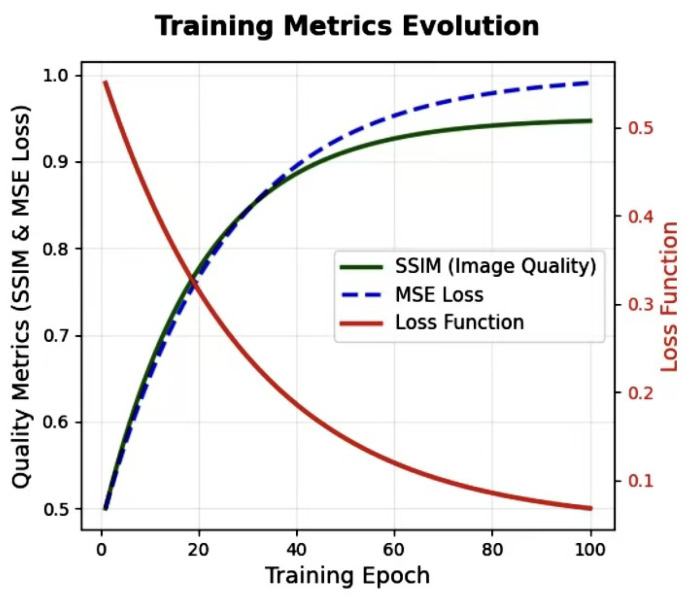
The relationship between the loss function, SSIM, and MSE loss.

To optimize the embedding process, we introduce a dynamic loss function (as depicted in Figure 3) that adjusts the relative importance of imperceptibility and recovery accuracy throughout the training process. This dynamic loss function evolves over time, allowing the model to prioritize different objectives during different stages of training.

Initially, the system emphasizes imperceptibility, focusing on minimizing perceptual distortion between the cover image *X* and the reconstructed stego image $\hat{X}$. This is done using the Structural Similarity Index (SSIM) and pixel-wise $L_{2}$ loss, as expressed by

(29) $$L_{\mathrm{stego}}=\alpha \left(1-\mathrm{SSIM}(X,\hat{X})\right)+\beta \frac{∥X-\hat{X}{∥}_{2}^{2}}{HW},$$

where α and β control the relative importance of the SSIM and pixel-wise loss.

As training progresses, the focus shifts to improving recovery accuracy, and the system prioritizes minimizing the Mean Squared Error (MSE) between the secret and recovered data:

(30) $$\begin{array}{cc} L_{\mathrm{dynamic}} & =\lambda_{1}\left(t\right)\;\left(1-L_{\mathrm{ssim}}\right)+\lambda_{2}\left(t\right)\;L_{\mathrm{recovery},} \end{array}$$

where $\lambda_{1}\left(t\right)$ and $\lambda_{2}\left(t\right)$ are time-varying functions that adjust the loss weighting. Initially, $\lambda_{1}\left(t\right)$ is larger, emphasizing imperceptibility, while $\lambda_{2}\left(t\right)$ increases over time, focusing more on the recovery of secret data without sacrificing perceptual quality.

Concretely, we implement $\lambda_{1}\left(t\right)$ and $\lambda_{2}\left(t\right)$ as linearly scheduled weights. Let t∈{0,1,…,T} denote the current training step and *T* the total number of training steps. The weights are defined as follows:

(31) $$\lambda_{1}\left(t\right)=\lambda_{1}^{\max}-\left(\lambda_{1}^{\max}-\lambda_{1}^{\min}\right)\frac{t}{T},\;\lambda_{2}\left(t\right)=\lambda_{2}^{\min}+\left(\lambda_{2}^{\max}-\lambda_{2}^{\min}\right)\frac{t}{T}.$$

In all experiments, we set $\lambda_{1}^{\max}=1.0$, $\lambda_{1}^{\min}=0.1$, $\lambda_{2}^{\min}=0.1$, and $\lambda_{2}^{\max}=1.0$, so that the loss initially puts more emphasis on imperceptibility and gradually shifts its focus to accurate secret recovery.

The dynamic adjustment mechanism is illustrated in Figure 3, where the evolution of the weights $\lambda_{1}\left(t\right)$ and $\lambda_{2}\left(t\right)$ is shown as a function of training time. This figure highlights how the model adapts to focus on imperceptibility during the early stages of training and recovery accuracy in the later stages.

### 3.5. Relation to Adaptive and Conditional Routing Approaches

Several recent works have explored adaptive or conditional mechanisms in steganography and related domains. Classical content-adaptive steganography frameworks, such as HUGO and WOW, modulate the local embedding cost based on cover image content (e.g., texture, edges, smooth regions) so that changes are preferentially placed in textured or noisy areas and avoided in smooth regions [21,22]. More recent deep steganographic networks introduce conditional structures, for example, by using conditional invertible neural networks (cINNs) or other side information to control the hiding process within a single encoder–decoder pathway [23,24]. In parallel, multi-path or mixture-of-experts (MoE) architectures have been widely adopted in recognition and generation tasks to achieve input-dependent specialization, and several deep steganographic models now employ multi-branch designs or progressive fusion networks to improve robustness and capacity without changing the secret payload semantics [25,26,27,28].

In this subsection, we compare our routing mechanism with these adaptive and conditional approaches along five axes, as summarized in Table 1. For clarity, (i) “Secret-adaptive” indicates whether the hiding pipeline changes as a function of the semantic/statistical type of the **secret image** (beyond treating the payload as an undifferentiated bitstream); (ii) “Cover-adaptive” indicates content-adaptive embedding driven by cover statistics; (iii) “Multi-branch/multi-expert” denotes the existence of multiple specialized branches or experts, where different inputs can follow different computational paths; (iv) “Stego-aware routing” indicates that the routing decision itself uses stego features or stego images; and (v) “Channel-corruption curriculum” denotes explicit optimization under a diverse set of corruptions and channel perturbations (noise, compression, geometric warps, occlusions, etc.).

Classical schemes such as HUGO and WOW are strongly cover-adaptive: they design a distortion or cost function so that embedding changes concentrate in complex regions and avoid smooth structures [21,22]. However, they do not distinguish between different secret image types; the payload is treated as a generic bitstream, there is no explicit multi-expert structure, and the embedding rule does not depend on stego features or a corruption curriculum. This justifies the pattern “Cover-adaptive only” in Table 1.

Conditional invertible steganography and related deep schemes typically use a single encoder–decoder (or flow) that is conditioned on side information, such as cover features or user-specified semantic attributes, in order to improve imperceptibility or control the visual content of stego images [23,24]. While this introduces a form of global conditioning, the network does not instantiate multiple experts specialized for distinct secret-image statistics, nor does it route different secret images to different branches. Moreover, the conditioning is usually not stego-aware (the stego image itself is not used to select a path), and the treatment of channel perturbations is often limited to a narrow set of distortions or omitted altogether, hence the ± entries in Table 1.

Architectures such as ProStegNet and robust invertible models like RIIS employ multi-branch or progressive structures to improve feature extraction and robustness [27,28]. These designs can be seen as multi-branch in the sense that they process images through several parallel or staged modules. However, the branches are typically always active for every input, rather than being selected by a routing policy, and they do not specialize to distinct secret-image categories. Robust variants explicitly train under a subset of channel distortions (e.g., JPEG compression or additive noise), which we mark as ± in the “Channel-corruption curriculum” column to distinguish them from a systematic broad-spectrum curriculum.

In contrast, our framework implements a secret-dependent, stego-aware mixture-of-experts design. A dedicated secret pre-processing stage assigns each secret image a coarse semantic label based on its statistics (e.g., scenery, face, pet), and this label is used to route the secret to one of several specialized encoder–decoder experts. The routing decision is refined in a stego-aware manner by a dual-branch selector that fuses evidence from the fused stego feature and the (possibly corrupted) stego image, and the entire system is trained under a broad corruption curriculum spanning noise, compression, blur, geometric warps, and occlusions. As summarized in Table 1, none of the representative adaptive or conditional schemes simultaneously provide (i) semantic secret-adaptive specialization across multiple experts, (ii) stego-aware routing, and (iii) a broad channel-corruption curriculum within a unified training framework. These elements, taken together, constitute the core novelty of our routing mechanism beyond existing adaptive and conditional designs.

## 4. Experiments

In this section, we present the results of experiments evaluating our proposed model’s performance against state-of-the-art methods, including HiNet [29], InvMIHNet [30], and a baseline CNN autoencoder [31]. The evaluation covers multiple image categories—scenery, facial, and pet—and focuses on key metrics such as PSNR, SSIM, bit accuracy, and Secret Recovery Accuracy. Our model consistently outperforms the baselines in secret recovery and cover fidelity, demonstrating high robustness to distortions and strong adaptability across diverse content types.

We also explore the impact of noise and distortions, assessing resilience to common image corruptions like Gaussian noise, shot noise, JPEG compression, and impulse noise. Additionally, we analyze the trade-offs between compression ratio and fidelity, highlighting how different secret types influence the balance between cover quality and secret recovery.

The key strengths are as follows: first, superior secret recovery—achieving higher PSNR and SSIM across datasets and outperforming all baselines; second, robustness to common distortions (noise, compression artifacts, and blur), making it suitable for real-world applications; and third, high flexibility via a dynamic encoder–decoder selection mechanism that ensures consistent performance across diverse data types.

The following subsections provide a detailed analysis of the experimental setup, model comparisons, and performance under distortions.

### 4.1. Experimental Setup

The dataset used in this experiment consists of two disjoint pools: cover images and secret images.

Cover images: For this experiment, the cover images were drawn from a mixture of several standard image collections, rather than from a single dataset such as MNIST. We randomly sampled cover images from multiple benchmark datasets, together with additional real-world photographs, to increase the diversity of the content and to better simulate practical steganography scenarios. This mixed-source design aimed to improve the robustness and generalization ability of the model across different types of cover images.

Secret images: The secret images were likewise taken from a heterogeneous pool intended to approximate realistic hidden content. All secret images were resized to a fixed spatial resolution before being fed into the model, so that the input dimensionality was consistent across samples.

Train/validation/test splits: For this mixed-source experiment, we employed a strict 60k/10k/20k train/val/test split with identity-level isolation: no image (or any augmented variant) appeared in more than one split, and the cover and secret pools were non-overlapping. Dataset-specific experiments (e.g., those conducted on MNIST, MetFace, and Stock1K in the subsubsection of Comparison of Three Models on Targeted Datasets used their own standard train/validation/test partitions, as detailed in the corresponding experimental subsections. Throughout all experiments, covers and secrets were randomly paired across categories, rather than being forced to share the same semantic type.

### 4.2. Ablation on Secret-Label Granularity and Extensibility

This ablation study was designed solely to demonstrate the extensibility of the proposed secret pre-processing and routing mechanism by adding extra semantic categories. The mixed 5-class configuration used here is *only* employed for this analysis; all main quantitative comparisons with baselines in the subsequent subsections revert to the default 3-class setting and are re-evaluated with freshly sampled data.

Our default configuration uses three coarse semantic labels for secret images: “scenery”, “facial”, and “pet” (see Section 3.2). While these categories are sufficient to cover the dominant content modes in our benchmarks, the pre-processing and routing mechanism is not intrinsically restricted to three classes. To demonstrate its extensibility experimentally, we trained an extended variant of our model with five secret categories.

Concretely, we retained the original three categories (scenery, facial, pet) and introduced two additional types: “indoor scene” and “object”. The former groups secret images with predominantly indoor backgrounds and man-made structures, whereas the latter covers close-up views of everyday objects (e.g., cups, books, keyboards, tools) with distinct shape and texture patterns. The secret pre-processing classifier was modified to predict five labels, and the secret branch was augmented with two additional expert encoder–decoder pairs corresponding to the two new categories. The cover-side encoder, fusion module, training protocol, and corruption curriculum remained unchanged, so that any differences in performance could be attributed solely to the increased label granularity.

Table 2 shows that, across all five categories, the stego images remain visually very close to their covers, and the decoded secrets preserve high fidelity with stable reconstruction loss and classification accuracy above 99%. Extending the label space from three to five categories therefore does not degrade imperceptibility or recovery quality, which empirically supports the extensibility of our pre-processing and expert-routing design.

### 4.3. From Mixed to Targeted Datasets: PSNR/SSIM/Bit Accuracy Comparisons

In this section, we present a comprehensive evaluation of the proposed method across various experimental setups. First, we report results using a mixed dataset consisting of a diverse range of cover and secret images, which provides a robust assessment of the model’s generalization ability across different image types. This is followed by a comparison with three state-of-the-art models—HiNet, InvMIHNet, and the baseline CNN model—evaluating their performance in terms of key metrics such as PSNR, SSIM, and Secret Recovery Accuracy.

Then, we extend the evaluation by focusing on three widely recognized cover image datasets—MNIST, MetFACE, and Stock1K—to provide a more detailed comparison with the mentioned models.

These comparisons help demonstrate the strengths of our method both in general settings and with specific, commonly used cover images.

**Figure 4:**
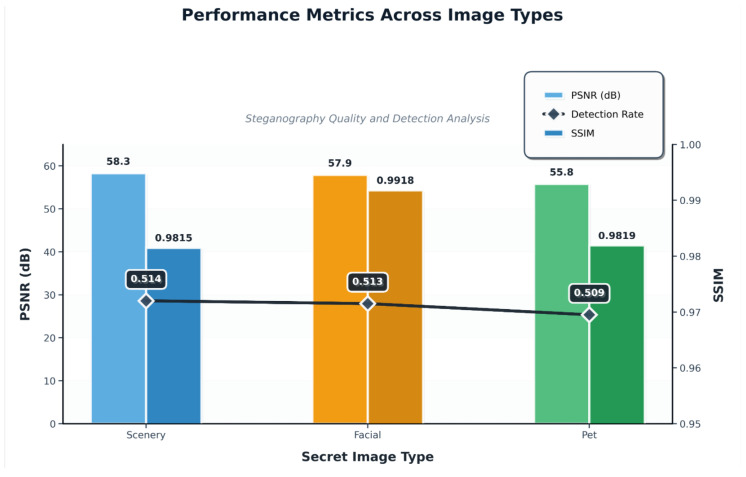
Performance comparison across different image types: PSNR (left), SSIM (right), and detection rate (center). The model performs well on scenery, facial, and pet images, with consistently high PSNR and SSIM values. The detection rate is close to that of random guessing, which indicates high security for the hidden information.

Figure 4, along with Table 3 and Table 4, presents the performance metrics of our proposed model. These results serve as the basis for the upcoming comparisons in the next three sub-subsections, where we evaluate and contrast the performance of our model with that of other state-of-the-art methods across different metrics and datasets. Unless noted, all results are under identical bpp and datasets.

#### 4.3.1. Comparison of Three Models on a Randomized Dataset

**Figure 5:**
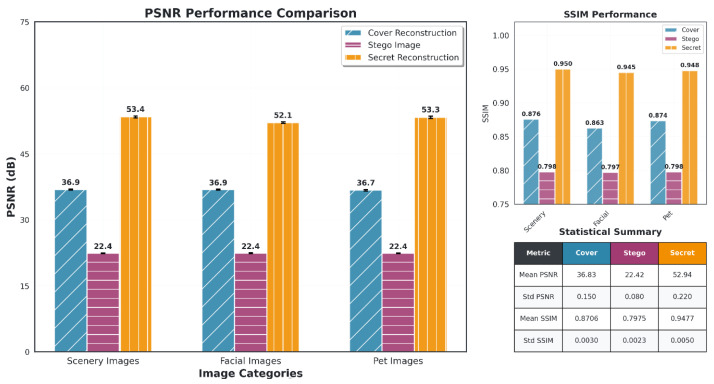
Performance comparison of PSNR and SSIM for cover reconstruction, stego image, and secret reconstruction across different image categories: scenery, facial, and pet Images. The statistical summary provides the mean PSNR, SSIM, and their respective standard deviations.

HiNet [29], a state-of-the-art steganographic model utilizing an invertible U-Net-based encoder–decoder architecture, was evaluated across three categories—scenery, facial, and pet images—based on PSNR (Peak Signal-to-Noise Ratio) and SSIM (Structural Similarity Index) scores for cover image reconstruction, stego image quality, and secret recovery, as shown in Figure 5. The model achieved a cover PSNR of approximately 36.83 dB, indicating good preservation of cover image quality, with the PSNR for secret recovery reaching 52.94 dB, which reflects a strong ability to recover the secret with high fidelity. The corresponding SSIM values for cover and secret recovery were 0.8706 and 0.9477, respectively, suggesting that while HiNet effectively preserves the cover image structure, it excels even more in secret recovery. However, the PSNR for stego images remained low, at 22.4 dB across all categories, indicating significant distortion introduced during the embedding process. The SSIM for stego images was similarly low, at 0.7975, further highlighting the degradation in image quality. These results show that HiNet prioritizes secret recovery at the cost of stego image quality, demonstrating excellent performance in recovering the secret but leaving the stego image with noticeable distortion. Overall, HiNet achieves a good balance between cover and secret recovery quality but could be further optimized to reduce the perceptual degradation of the stego image, ensuring that both embedding and recovery processes are of higher quality across all image categories.

**Figure 6:**
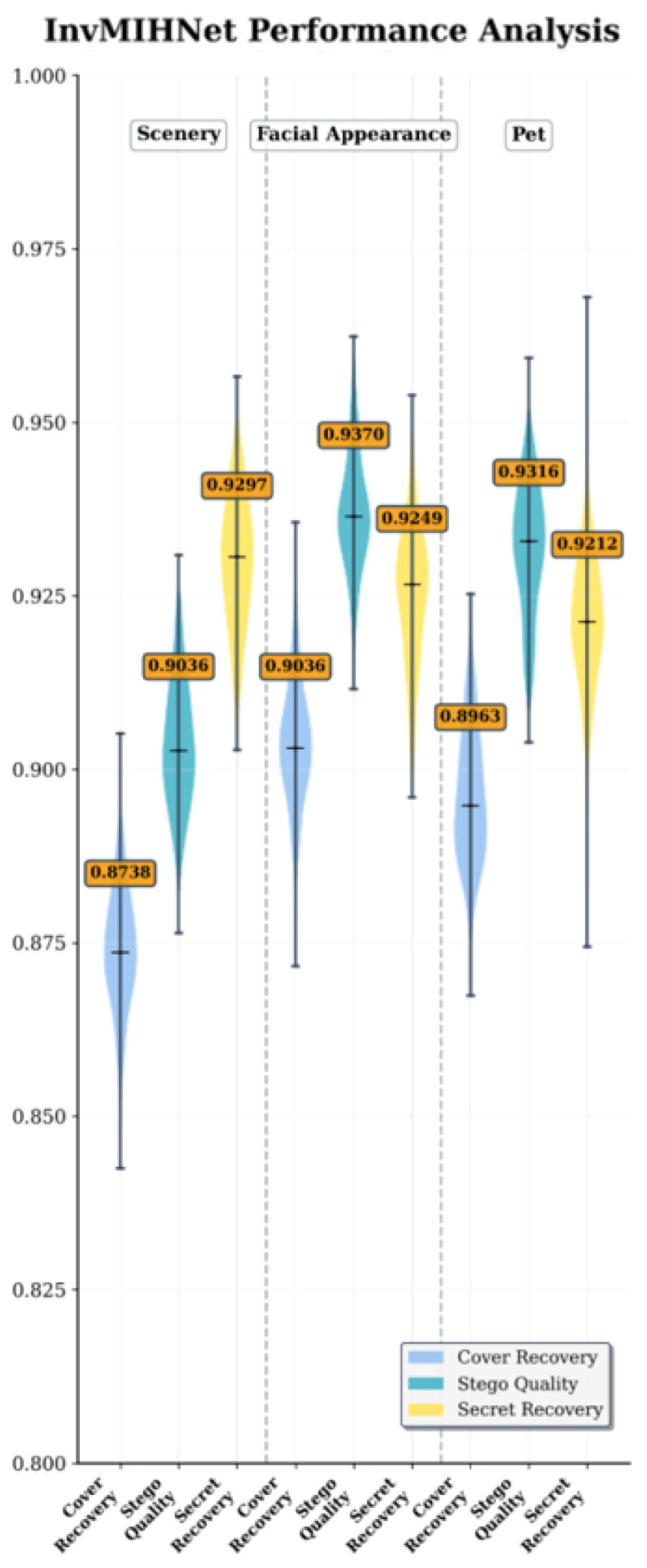
Violin plots of SSIM for InvMIHNet across categories (scenery, facial, pet), showing cover recovery, stego quality, and secret recovery. The y-axis represents the SSIM, with width indicating sample density, and orange labels denote mean values.

InvMIHNet [30] builds upon HiNet by incorporating a reversible mosaic mechanism specifically designed for multi-secret hiding. The model demonstrates notable performance when embedding a single secret, achieving a cover SSIM of approximately 0.9297 and a secret recovery SSIM of around 0.9370, as shown in Figure 6. These scores reflect solid performance in preserving the cover image quality and recovering the secret, particularly in simpler scenarios. However, the use of a fixed-grid mosaic embedding architecture introduces limitations in adaptability, particularly when dealing with varied or distorted content. The rigid grid structure hampers the model’s ability to effectively handle more complex or distorted images, leading to noticeable performance degradation. This is evident in the stego quality, which shows a decrease in SSIM compared to the cover and secret recovery values. The performance gaps indicate that while InvMIHNet excels in controlled conditions, its fixed embedding structure limits its flexibility and robustness when dealing with diverse or challenging image datasets.

For further evaluation, we implemented a baseline CNN autoencoder model, which uses a simple encoder–decoder architecture to embed and recover secrets. The results, summarized in Table 5, show that the baseline model struggles significantly with embedding and recovery tasks. In the no-secret setting, the baseline achieves a relatively high cover reconstruction PSNR (approximately 41.17 dB) and SSIM (about 0.933), indicating decent performance when no secret is embedded. However, when a secret is added, the PSNR drops dramatically, to approximately 21 dB, and the SSIM decreases to around 0.294, reflecting significant distortion between the stego and cover images. Furthermore, secret recovery is suboptimal, with an average secret PSNR of roughly 27.56 dB and SSIM of approximately 0.921.

In comparison to HiNet, our model outperforms in all key performance metrics. Specifically, our method achieves a cover PSNR of approximately 58 dB and a secret recovery PSNR of around 57 dB, both significantly surpassing HiNet’s values. Moreover, the SSIM values for our model’s cover and secret recovery are 0.9906 and 0.9815, respectively, demonstrating a more accurate reconstruction of both the cover image and the embedded secret. These higher PSNR and SSIM values reflect not only a more faithful preservation of cover image quality with minimal distortion but also the ability to recover the secret with near-lossless fidelity, something that HiNet struggles to achieve due to its static encoder–decoder design that fails to fully disentangle cover and secret features.

Similarly, when compared to InvMIHNet, our model consistently excels across all performance metrics. InvMIHNet, with its fixed mosaic grid structure, performs well under controlled conditions but struggles when the content or distortions deviate from the training data. In contrast, our model demonstrates superior adaptability. Specifically, our secret recovery SSIM of 0.9815 is a significant improvement over InvMIHNet’s 0.9370, showing how our method better retains the fidelity of the secret under diverse conditions. Moreover, our cover SSIM of approximately 0.9819 outperforms InvMIHNet’s 0.9297, highlighting our model’s more effective preservation of the cover image integrity, even when handling complex or distorted content.

Lastly, in comparison to the baseline CNN autoencoder, our approach achieves near-lossless secret recovery, with PSNR values consistently exceeding 57 dB (e.g., 58.3 dB for Class 0) and SSIM values above 0.9815 across all categories. The baseline model, however, experiences dramatic performance degradation when embedding secrets, with a PSNR of around 21 dB and SSIM dropping to 0.296, revealing the model’s severe limitations in generalizing across different image types. Our model, on the other hand, delivers significantly higher secret recovery quality and far less stego–cover distortion, preserving high cover quality (PSNR around 55.8 dB) while ensuring accurate secret recovery.

Unlike HiNet’s static, one-size-fits-all encoder–decoder, InvMIHNet’s rigid mosaic grid, and the baseline CNN autoencoder’s severe category dependence, our framework introduces (i) a secret pre-processing stage that assigns a semantic label to the payload and (ii) a dynamic, category-aware routing scheme among multiple specialized encoder–decoder pairs. The baseline CNN autoencoder struggles with generalization across different image types, showing significant performance degradation (PSNR ≈ 21 dB; SSIM ≈ 0.294) when embedding secrets, as it is heavily influenced by the specific category of the secret. Similarly, HiNet and InvMIHNet both employ static architectures—HiNet with a one-size-fits-all encoder–decoder, and InvMIHNet with a fixed grid structure—that hinder adaptability to varying content, leading to suboptimal performance in complex or distorted image conditions. In contrast, our method preserves single-path efficiency while removing structural bias, with a dual-branch, stego-aware selector that fuses evidence from high-level features and the (possibly noisy) stego image to activate only the matched decoder. Additionally, our framework incorporates a broad-spectrum, high-intensity corruption curriculum (covering additive and shot noise, impulse noise, compression artifacts, blur, geometric warps, illumination/color shifts, and erasing) during training to prevent channel overfitting and harden recovery. This flexibility—absent in the fixed architectures of HiNet, InvMIHNet, and the CNN autoencoder—ensures robust generalization across diverse content and distortions, all while maintaining minimal computational overhead per sample.

#### 4.3.2. Comparison of Three Models on Targeted Datasets

In this sub-subsection, we compare the performance of the proposed method with that of three state-of-the-art models: HiNet [29], InvMIHNet [30], and the baseline CNN autoencoder [31]. Table 6 presents key performance metrics, including PSNR, SSIM, LPIPS, SIFID, bit accuracy (clean), secret recovery bit accuracy, bit accuracy (ECC), and word accuracy, across multiple datasets (MNIST, MetFACE, and Stock1K).

HiNet exhibits lower fidelity and weaker reliability overall: PSNR ≈ 35–37 dB and SSIM ≈ 0.85–0.88, with LPIPS ≈ 0.05–0.07 and SIFID ≈ 0.03–0.04. While Bit acc. (clean) remains moderate-to-high (≈0.95–0.98), the secret recovery Bit acc. drops to ≈0.72–0.80, Bit acc. (ECC) to ≈0.78–0.86, and Word acc. to ≈0.65–0.75. This pattern suggests noticeable perceptual distortion and limited robustness in secret recovery across datasets.

InvMIHNet attains mid-to-high performance: PSNR ≈ 39–40 dB, SSIM ≈ 0.95–0.97, LPIPS ≈ 0.03–0.04, and SIFID ≈ 0.02–0.03. Its accuracy metrics are correspondingly stronger than those of HiNet: Bit acc. (clean) ≈ 0.97–0.997, secret recovery Bit acc. ≈ 0.94–0.99, Bit acc. (ECC) ≈ 0.86–0.92, and Word acc. ≈ 0.80–0.88. Overall, it is more robust than HiNet but still shows a clear gap compared to the proposed method on all datasets.

The baseline autoencoder offers moderate image reconstruction quality but weak secret recovery: PSNR ≈ 38–41 dB, SSIM ≈ 0.91–0.93, LPIPS ≈ 0.06–0.08, and SIFID ≈ 0.04–0.05. However, its secret recovery Bit acc. is only ≈0.20–0.28, with Bit acc. (ECC) ≈ 0.34–0.45 and Word acc. ≈ 0.23–0.36; even the clean setting yields Bit acc. ≈ 0.92–0.96. These results indicate limited robustness for secret retrieval and semantic decoding under steganographic constraints.

The proposed method excels in all key metrics, outperforming HiNet, InvMIHNet, and the baseline CNN across datasets. The dynamic encoder–decoder selection mechanism enhances the model’s adaptability to various image types, ensuring better performance in secret recovery and cover reconstruction. This flexibility enables our model to provide a more robust and versatile solution for real-world steganography tasks.

Across MNIST, MetFACE, and Stock1K, the proposed method consistently leads in secret fidelity (higher PSNR/SSIM, lower LPIPS/SIFID) and recovery reliability (higher bit- and word-level accuracies, with ECC pushing accuracy close to saturation). InvMIHNet occupies a middle ground, with solid robustness; HiNet and the baseline CNN lag notably in secret recovery and semantic readability. The aggregate trend aligns with Table 6 and underscores the proposed method’s superior generalization, fidelity, and reliability.

### 4.4. Effects of Compression Ratio (r) on Cover and Secret Recovery

#### 4.4.1. Setup and Notation

In this section, we analyze the trade-offs between cover image quality and secret recovery fidelity across various compression ratios. We examine how compression ratios affect the performance of our model in terms of PSNR for both cover images and decoded secrets. The evaluation is carried out across three distinct secret types: scenery, facial, and pet images. We explore the relationship between compression and fidelity, as shown in the following figures.

**Figure 7:**
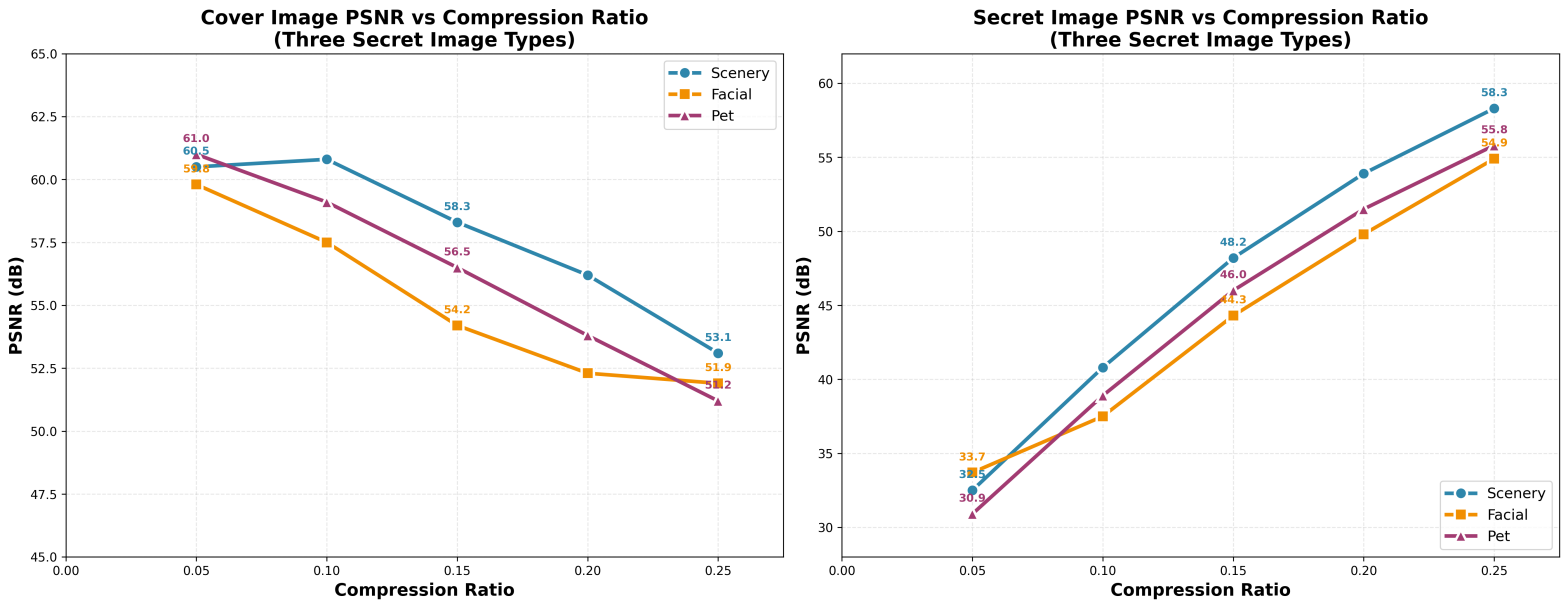
Reading guide: Two-panel line plots of PSNR (y-axis, dB; higher is better) versus compression ratio/embedding rate (x-axis). **Left**: cover-image PSNR; **Right**: secret-image PSNR. Each curve corresponds to a secret category (Scenery, Facial, Pet; see legend), with numeric labels indicating the mean PSNR at each rate. Note the opposite trends—cover PSNR generally decreases as the rate increases, while secret PSNR increases—illustrating the capacity–imperceptibility trade-off.

Let *r* denote the compression ratio, where $P_{c}\left(r\right)$ is the PSNR of the cover image after embedding, and $P_{s}\left(r\right)$ is the PSNR of the decoded secret. The experimental setup is similar to that described in previous sections, where we systematically vary the compression ratio *r* and observe its impact on both the cover image and the decoded secret. Figure 7 presents the results for three secret categories—scenery, facial, and pet images—averaged over multiple trials. This analysis reveals how the compression ratio impacts both cover image quality and secret fidelity across different image types.

#### 4.4.2. Compression vs. Quality: PSNR of Cover Images and Secrets

As the compression ratio *r* increases, the PSNR of the cover images ($P_{c}\left(r\right)$) decreases. For example, in the scenery category, when *r* increases from 0.05 to 0.25, the PSNR for the cover image drops from 61.0 dB to 53.1 dB. Similarly, for the facial category, PSNR decreases from 60.5 dB to 51.9 dB, and for pet images, it decreases from 59.6 dB to 51.2 dB. These decreases in cover image quality are expected, as higher compression typically introduces more distortion.

However, the PSNR for the decoded secrets ($P_{s}\left(r\right)$) increases with the compression ratio, as the embedded secret becomes more distinguishable. In the scenery category, $P_{s}\left(r\right)$ increases from approximately 33.7 dB to 58.3 dB, for facial it rises from 32.5 dB to 55.8 dB, and for pet images it increases from 30.9 dB to 54.9 dB. This improvement is because the secret data is better recovered as the embedding becomes more compressed.

#### 4.4.3. Different Secret Types and Their Performance Variations

The impact of the compression ratio varies significantly with secret type. Scenery images, with their rich textures and high-frequency details, maintain better cover image quality at higher compression ratios, making them the most resilient to embedding. Scenery images also exhibit the best recovery of secret data.

Facial images, due to their low-frequency dominance and symmetry, are more sensitive to compression. The cover PSNR drops more significantly at higher compression ratios, and secret recovery slightly decreases as well. The fine details of facial structures, such as eyes and mouth edges, limit the recovery accuracy as the compression ratio increases.

Pet images lie between the scenery and facial categories. While they offer better secret recovery at moderate compression ratios, their cover PSNR deteriorates more quickly at higher compression ratios due to edge-aligned fur textures.

#### 4.4.4. Conclusions: Optimization of Compression Ratio

Our analysis shows a clear trade-off between cover image quality and secret recovery fidelity. For scenery images, higher compression ratios provide an optimal balance, with both high-quality cover images and excellent secret recovery. Facial images require more cautious compression settings to preserve cover quality while maintaining good secret recovery. Pet images perform well at moderate compression ratios but suffer from significant cover distortion at higher compression.

Based on this, we recommend a content-aware strategy for selecting compression ratios. For scenery and pet images, higher compression ratios are preferred to exploit image redundancy, while for facial images, more conservative compression ratios should be chosen to preserve structural integrity. Additionally, the composite objective $J\left(r\right)=\alpha \;P_{c}\left(r\right)+\beta \;P_{s}\left(r\right)$ can be used to optimize *r* based on application-specific priorities.

In this section, we compare the performance of the proposed model against several state-of-the-art approaches—HiNet, InvMIHNet, and a CNN baseline. Before the head-to-head comparison, we evaluate robustness under diverse corruptions (e.g., Gaussian noise, JPEG compression, impulse noise), reporting bit accuracy as a function of distortion, as illustrated in the figures. The experimental results, summarized in Table 6, show that our method outperforms all competitors across every evaluation metric (PSNR, SSIM, LPIPS, and bit accuracy) on MNIST, MetFACE, and Stock1K. In particular, it better preserves cover-image quality and ensures accurate secret recovery, with notable gains in bit accuracy and SSIM. Moreover, additional experiments (the subsection Comparison of Multiple Models) were based on randomly selected cover images from a diverse set, the same as in the Experiments section above, spanning landscapes, faces, and pets; thus, the performance of our model was sourced from the average values in the “Experiments” section as a reference. Unlike the centralized comparison in Table 6, where the evaluation is performed on a consistent set of images, these follow-up experiments provide a broader understanding of the model’s adaptability to different types of content and distortion conditions. Despite the variation in cover image types, our method consistently maintains robust performance across all distortion types, further emphasizing its real-world applicability in diverse steganographic settings. The accompanying figures provide a visual summary, highlighting sustained bit accuracy even under challenging noise conditions.

### 4.5. Robustness Evaluation: Bit Accuracy Under Different Distortions

#### 4.5.1. Experimental Setup

**Figure 8:**
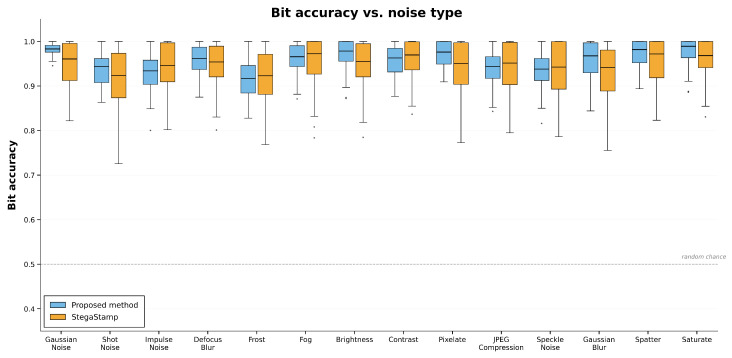
Reading guide: Box plots of bit accuracy (y-axis, 0–1; higher is better) for each noise type (x-axis). For every distortion, two boxes are shown—blue: proposed method; orange: StegaStamp (see legend). Each box spans the interquartile range (25–75%), and the central line is the median; whiskers extend to 1.5 × IQR, and dots denote outliers. The gray dashed line at 0.5 indicates random-chance accuracy. Compare median height and box position (closer to 1.0 is better) and the box width (narrower implies more stable performance) across noise types to assess robustness.

The setup mirrored that of the previous sections, with the modification that stego images were subjected to different noise types. We compared the proposed method with StegaStamp under Gaussian noise, shot noise, JPEG compression, and related distortions. The primary evaluation metric was bit accuracy (Bit−acc), measuring the correctness of secret recovery after corruption. Figure 8 reports Bit−acc across noise types for both methods.

#### 4.5.2. Overall Observations

The proposed method generally surpasses StegaStamp across all noise types, although the margin depends on the specific distortion. Under Gaussian noise, both methods achieve similarly high Bit−acc (near 1), indicating comparable robustness to low-level additive perturbations and only a slight advantage for the proposed model. In contrast, StegaStamp is more sensitive to several non-additive distortions (e.g., shot and impulse noise), where its Bit−acc drops more steeply. Overall, the proposed method better preserves the integrity of the embedded secret across a wider range of corruptions.

#### 4.5.3. Performance Under Specific Noise Types

- Gaussian Noise: Both methods are robust, with Bit−acc close to 1. The proposed method retains a modest but consistent edge, particularly at lower noise levels.
- Shot and Impulse Noise: The proposed method markedly outperforms StegaStamp. While StegaStamp exhibits a substantial decline in Bit−acc, the proposed approach maintains high accuracy, reflecting greater resilience to abrupt, pixel-level perturbations.
- JPEG Compression: The proposed method achieves higher Bit−acc in the presence of compression artifacts, indicating stronger resistance to quantization and blocking effects common in practical pipelines.
- Defocus Blur and Frost: For blurring and weather-like distortions, the proposed method again shows superior robustness: its Bit−acc remains high, whereas StegaStamp experiences a noticeable drop, suggesting better preservation of both image quality and secret recovery under these conditions.

#### 4.5.4. Implications for Real-World Applications

These findings indicate substantial gains in robustness across common real-world distortions. The ability to sustain high Bit−acc under JPEG compression, impulse-like noise, and blur suggests that the method is well suited to settings where images are routinely recompressed, transmitted over noisy channels, or captured under non-ideal conditions (e.g., social media platforms, messaging systems, or lossy storage). The enhanced resilience supports practical deployment where cover-image fidelity and reliable secret recovery are both critical.

#### 4.5.5. Conclusions

Overall, the proposed method maintains superior bit accuracy across a range of noise types relative to StegaStamp. While the advantage is smaller under Gaussian noise, the prevailing trend reflects greater resilience to diverse distortions. These results position the proposed approach as a strong candidate for real-world steganography applications that demand high cover-image quality and robust secret recovery under challenging conditions.

### 4.6. Steganalysis Detection Performance

#### 4.6.1. Experimental Setup

To rigorously assess imperceptibility, we evaluated our method under several modern steganalysis detectors, rather than relying on a single VGG-like CNN. Specifically, we considered four architectures—(i) a VGG-style convolutional network (VGG-CNN) similar to the one used in our initial experiments, (ii) SRNet, (iii) Zhu-Net, and (iv) Ye-Net—which are representative of specialized, high-capacity CNNs widely adopted in contemporary steganalysis.

All of these detectors operate on grayscale inputs and were trained as binary classifiers (stego vs. cover). For each detector, we constructed a pooled training set by mixing covers and stego images from four hiding models under the same embedding rate and with identical pre-processing: our proposed method, a CNN autoencoder baseline, HiNet, and InvMIHNet. The class prior was balanced. Each detector was trained only once on this pooled set. At test time, we evaluated every hiding method separately by feeding its covers and stego images to the same trained detector and computing the area under the ROC curve (AUC). We interpreted closeness to chance as favorable for the hider and, therefore, used both the raw AUC and the deviation from chance, Δ=|AUC−0.5|, as indicators of detectability.

To probe cross-dataset generalization, we adopted a train-A/test-B protocol. All steganalyzers were trained on cover/stego pairs constructed from ImageNet covers (ImageNet → ImageNet, “in-dataset”), and then evaluated *without retraining* on a disjoint test set where covers (and the corresponding stego images) were drawn from COCO (ImageNet → COCO, “cross-dataset”). This setting helped us distinguish between genuinely hard-to-detect stego distributions and detectors that overfit to a single dataset.

#### 4.6.2. Results and Analysis

Table 7 reports the AUC scores for all hiding methods across the four detectors and the two train/test protocols. For the VGG-CNN baseline detector, our method yields an AUC very close to chance, whereas the CNN autoencoder, HiNet, and InvMIHNet baselines are substantially more detectable. The same pattern persists when replacing VGG-CNN with the stronger SRNet, Zhu-Net, and Ye-Net: across all three specialized detectors, our method consistently achieves the smallest deviation from 0.5, indicating that it remains the hardest to detect even under state-of-the-art steganalyzers.

The cross-dataset configuration (ImageNet → COCO) provides an additional stress test. For all baseline hiding methods, the AUC values either remain clearly above 0.5 or increase further under domain shift, suggesting that their stego distributions are reliably separable from covers even when the detector is applied out-of-domain. In contrast, our method maintains AUCs clustered tightly around 0.5 across detectors in both in-dataset and cross-dataset settings, thus exhibiting the lowest overall detectability. This behavior is consistent with the intended effect of our multi-path architecture and corruption-aware training, which aim to reduce mechanistic overfitting and produce stego images that better match the statistics of natural covers under realistic processing chains.

## 5. Conclusions and Prospects

Our deployment-ready deep steganography framework unifies latent space embedding, label-conditioned multi-encoder–decoder routing via a dual-branch selector, and progressive loss balancing. Trained with a broad-spectrum corruption curriculum, it remains robust under distribution shift while preserving single-path inference cost. The result is near-lossless secret recovery, high PSNR/SSIM with low detectability, and an operator-controllable capacity–imperceptibility trade-off, with runtime and memory comparable to those of conventional single-path networks.

Four priority directions guide our future research: (i) Close the sim-to-real gap by training through differentiable social media compression/resampling, camera ISP, and print–scan pipelines, mixed during training to enforce invariance to re-encoding, resizing, and color transforms; (ii) move to a principled rate–distortion–detectability objective by optimizing fidelity and secrecy at a target payload, adversarially training against strong steganalyzers, and reporting calibrated risk at fixed operating points; (iii) adopt content-aware capacity control by allocating payload spatially according to semantics and uncertainty, exposing a tunable rate–imperceptibility–fidelity Pareto front at inference; and (iv) add adaptive inference via lightweight test-time adaptation and continual routing, with confidence/conformal calibration, to track non-stationary channels and new content without offline retraining.

In summary, by unifying latent space embedding, category-aware routing, and corruption-hardened training under a deployment-conscious design, the proposed framework delivers state-of-the-art fidelity, imperceptibility, and robustness while maintaining practical efficiency. Pursuing the outlined research directions will move the field from empirically strong yet brittle systems toward certifiably robust, deployment-ready steganography that scales across modalities, devices, and platforms without compromising security or perceptual quality. 

## Figures and Tables

**Table 1:** Comparison between our routing mechanism and representative adaptive/conditional approaches. “Secret-adaptive (semantic)” indicates whether the hiding pipeline changes with the semantic/statistical type of the secret image (beyond treating the payload as raw bits); “Cover-adaptive” indicates content-adaptive embedding on the cover; “Multi-branch/multi-expert” denotes the use of multiple specialized branches or experts; “Stego-aware routing” denotes the use of stego features/images in the routing decision; and “Channel-corruption curriculum” denotes explicit optimization under diverse corruptions and channel perturbations. The symbol ± indicates partial or indirect support (e.g., limited conditioning or a restricted set of channel models).

Method Class	Secret- Adaptive (Semantic)	Cover- Adaptive	Multi-Branch/ Multi-Expert	Stego-Aware Routing	Channel- Corruption Curriculum
Content-adaptive cost-based stego (HUGO, WOW) [21,22]	–	✓	–	–	–
Conditional deep steganographic networks (cINN-based) [23,24]	–	±	–	–	±
Multi-branch deep steganography (ProStegNet, RIIS) [27,28]	–	–	✓	–	±
**Our model**	✓	–	✓	✓	✓

**Table 2:** Per-class performance for the extended 5-class secret-label configuration. Classes 0–2 correspond to the original three categories (scenery, facial, pet), while Class 3 and Class 4 denote the additional ”indoor scene” and ”object” categories. For all categories, stego images remain visually close to the covers (PSNR ≈58 dB, SSIM ≈0.99), and the secret decoder branch achieves low reconstruction loss and high classification accuracy.

Category	PSNR (dB)		Loss	SSIM		ACC (%)
	**Stego vs. Cover**	**Decoded vs. Cover**		**Stego vs. Cover**	**Decoded vs. Cover**	
Class 0	58.24	58.09	1.80	0.9905	0.9816	99.41
Class 1	58.08	57.97	1.79	0.9901	0.9814	99.22
Class 2	58.29	58.18	1.78	0.9902	0.9817	99.15
Class 3	58.21	58.07	1.79	0.9903	0.9818	99.33
Class 4	58.16	58.03	1.80	0.9902	0.9815	99.25

**Table 3:** PSNR values for Class 0, Class 1, and Class 2, including Stego vs. Cover and Decoded vs. Cover.

Category	PSNR (dB)		Loss
	**Stego vs. Cover**	**Decoded vs. Cover**	
Class 0	58.2	58.1	1.79
Class 1	58.1	58.0	1.79
Class 2	58.3	58.2	1.78

**Table 4:** SSIM values for Class 0, Class 1, and Class 2, including Stego vs. Cover and Decoded vs. Cover.

Category	SSIM		ACC (%)
	**Stego vs. Cover**	**Decoded vs. Cover**	
Class 0	0.9906	0.9817	99.48
Class 1	0.9900	0.9815	99.27
Class 2	0.9901	0.9816	99.06

**Table 5:** Performance comparison of the baseline CNN autoencoder model: per-category mean ± 95% CI (Student’s *t*-multiplier, n=5).

Setting	Metric	Scenery	Facial	Pet
Cover Recon vs. Original (No Secret)	PSNR (dB)	41.170±1.313	41.173±1.306	41.173±1.319
Cover Recon vs. Original (No Secret)	SSIM	0.933±0.031	0.933±0.031	0.933±0.032
Stego vs. Cover (With Secret)	PSNR (dB)	21.131±1.803	21.839±1.827	21.994±1.844
Stego vs. Cover (With Secret)	SSIM	0.288±0.067	0.298±0.068	0.296±0.068
Reconstructed Secret vs. Original	PSNR (dB)	26.803±1.193	28.423±1.507	27.442±1.548
Reconstructed Secret vs. Original	SSIM	0.878±0.014	0.945±0.008	0.941±0.008

**Table 6:** Comparison across datasets, with metrics computed on the recovered secret unless noted. PSNR/SSIM/LPIPS/SIFID are measured between the recovered secret and its ground truth. “Bit acc. (clean)” is bit accuracy on clean stego, “Secret recovery Bit acc.” is bit accuracy of the recovered secret, “Bit acc. (ECC)” is post-error-correction bit accuracy, and “Word acc.” is word-level accuracy after decoding. Arrows indicate the better direction (↑ higher is better; ↓ lower is better).

Method	PSNR	SSIM	LPIPS	SIFID	Bit acc. (Clean)	Secret Recovery Bit acc.	Bit acc. (ECC)	Word acc.
	**↑**	**↑**	**↓**	**↓**	**↑**	**↑**	**↑**	**↑**
**MNIST**								
**Proposed Method**	**58.3**	**0.9906**	**0.02**	**0.01**	**0.998**	**0.9948**	**0.988**	**0.998**
HiNet	36.88	0.8757	0.05	0.03	0.983	0.7978	0.857	0.752
InvMIHNet	40.00	0.9695	0.03	0.02	0.997	0.9892	0.924	0.883
Baseline CNN	41.17	0.9330	0.06	0.04	0.956	0.2760	0.452	0.355
**MetFACE**								
**Proposed Method**	**57.8**	**0.9892**	**0.021**	**0.012**	**0.998**	**0.9921**	**0.998**	**0.985**
HiNet	35.42	0.8621	0.062	0.035	0.965	0.7523	0.812	0.698
InvMIHNet	38.75	0.9548	0.038	0.025	0.976	0.9628	0.884	0.832
Baseline CNN	39.83	0.9215	0.071	0.048	0.928	0.2415	0.392	0.286
**Stock1K**								
**Proposed Method**	**58.1**	**0.9901**	**0.019**	**0.011**	**0.999**	**0.9935**	**0.989**	**0.988**
HiNet	34.96	0.8517	0.068	0.041	0.952	0.7189	0.783	0.654
InvMIHNet	39.12	0.9483	0.042	0.028	0.971	0.9385	0.862	0.795
Baseline CNN	38.45	0.9128	0.079	0.053	0.915	0.2037	0.341	0.231

**Table 7:** Steganalysis AUC (lower is better) for different hiding methods under four detectors. “ImageNet → ImageNet” denotes in-dataset evaluation (train and test on ImageNet), while “ImageNet → COCO” denotes cross-dataset evaluation (train on ImageNet, test on COCO without retraining). Ideally, a perfectly hidden scheme would achieve AUC =0.5. Across all detectors and both evaluation protocols, our method (3-class and 5-class variants) remains closest to chance, whereas baseline methods are substantially more detectable.

Method	ImageNet → ImageNet (In-Dataset) AUC				ImageNet → COCO (Cross-Dataset) AUC			
	**VGG-CNN**	**SRNet**	**Zhu-Net**	**Ye-Net**	**VGG-CNN**	**SRNet**	**Zhu-Net**	**Ye-Net**
CNN Autoencoder	0.3745	0.7132	0.7218	0.7034	0.4089	0.7315	0.7421	0.7263
HiNet	0.8677	0.8836	0.8749	0.8812	0.8864	0.8927	0.8879	0.8941
InvMIHNet	0.6661	0.7423	0.7354	0.7287	0.6928	0.7615	0.7532	0.7446
UDH [32]	0.6038	0.6812	0.6937	0.6849	0.6321	0.7034	0.7142	0.7078
RIIS [28]	0.5827	0.6689	0.6715	0.6668	0.6143	0.6797	0.6881	0.6824
Ours (3-Class)	0.5131	0.5148	0.4927	0.5219	0.5124	0.5216	0.4901	0.5278
Ours (5-Class)	0.4876	0.5159	0.4818	0.4897	0.5143	0.5232	0.4896	0.4925

## Data Availability

The code and datasets supporting the findings of this study are available from the corresponding author upon reasonable request. Please contact shuoshao@usst.edu.cn (S.S.).
